# The Cell Wall Polysaccharides Biosynthesis in Seaweeds: A Molecular Perspective

**DOI:** 10.3389/fpls.2022.902823

**Published:** 2022-05-10

**Authors:** Zhanru Shao, Delin Duan

**Affiliations:** ^1^CAS and Shandong Province Key Laboratory of Experimental Marine Biology, Center for Ocean Mega-Science, Institute of Oceanology, Chinese Academy of Sciences, Qingdao, China; ^2^Laboratory for Marine Biology and Biotechnology, Pilot National Laboratory for Marine Science and Technology (Qingdao), Qingdao, China; ^3^State Key Laboratory of Bioactive Seaweed Substances, Qingdao Bright Moon Seaweed Group Co., Ltd., Qingdao, China

**Keywords:** cell wall polysaccharide, gene mining, mannuronan C-5 epimerase, carbohydrate sulfotransferase, biosynthesis

## Abstract

Cell wall polysaccharides (CWPS) of seaweeds play crucial roles in mechanical shear resistance, cell-cell adhesion and the interactions with changeable marine environments. They have diverse applications in food, cosmetics, agriculture, pharmaceuticals and therapeutics. The recent boost of multi-omics sequence analysis has rapidly progressed the mining of presumed genes encoding enzymes involved in CWPS biosynthesis pathways. In this review, we summarize the biosynthetic pathways of alginate, fucoidan, agar, carrageenan and ulvan in seaweeds referred to the literatures on published genomes and biochemical characterization of encoded enzymes. Some transcriptomic data were briefly reported to discuss the correlation between gene expression levels and CWPS contents. Mannuronan C-5 epimerase (MC5E) and carbohydrate sulfotransferase (CST) are crucial enzymes for alginate and sulfated CWPS, respectively. Nonetheless, most CWPS-relevant genes were merely investigated by gene mining and phylogenetic analysis. We offer an integrative view of CWPS biosynthesis from a molecular perspective and discuss about the underlying regulation mechanism. However, a clear understanding of the relationship between chemical structure and bioactivities of CWPS is limited, and reverse genetic manipulation and effective gene editing tools need to be developed in future.

## Introduction

Seaweeds (macroalgae) cover a wide group of algal phyla, and so far about 72,500 known species exist in diversified habitats (Guiry, 2012). Seaweeds, a potential climate change solution, have very important ecological roles in the ocean, serving as the base of the marine food chain and the vital force of marine carbon fixation and sequestration (Campbell et al., 2019; Ortega et al., 2019; Yong et al., 2022). Seaweeds contribute nearly 30% of the world aquaculture production (Cai et al., 2021). So far, the annual production of world aquatic algae increased over 60 times from 0.56 million tons in 1950 to 35.82 million tons in 2019, with 99.84% from seaweeds and 97% contributed by cultivation (FAO, 2021). The value of the commercial seaweed market worldwide in 2028 was estimated to exceed 24.9 billion U.S. dollars (Shahbandeh, 2021). Seaweeds produce unsaturated fatty acids, minerals, vitamins, phycobiliproteins and polysaccharides for diverse applications in food, animal feed, cosmetics and pharmaceuticals (Vincent et al., 2020). These make seaweeds attract increasing interests in science and industry (Leandro et al., 2020).

Seaweeds are traditionally grouped into three distinct classes based on pigmentation: brown (Ochrophyta), green (Chlorophyta) and red (Rhodophyta) algae (Guiry and Guiry, 2014). They can produce unique carbohydrates due to the complexity of their evolutionary history and habitats (Baldauf, 2008; Coelho and Cock, 2020). The average content of polysaccharides in seaweeds is around 50% (dry weight) and can reach up to 76% (Rioux and Turgeon, 2015). In most taxa of seaweeds, cell walls consist of microfibrillar networks embedded in matrices of diverse polysaccharides and proteins (Domozych, 2016). Cell wall polysaccharides (CWPS) contribute significantly to mechanical shear resistance, cell-cell adhesion, reproduction and morphogenesis, enhanced flexibility and interactions with changeable marine environments. The environmental benefits, ecosystem services and health contributions of seaweeds are inseparable from the biosynthesis of CWPS. Generally, CWPS constitute the largest source of annual renewable biomass on Earth (Domozych, 2016). Specifically, these polysaccharides designate as alginate and fucoidan in brown, agar and carrageenan in red and ulvan in green seaweeds. As macromolecule materials, the structure of these polysaccharides relies on the seaweed species, growth seasons, harvest locations and maturity and so forth. Previously, the extraction, structural determination, activity and function were extensively investigated (Kidgell et al., 2019; Rhein-Knudsen and Meyer, 2021). However, the enzyme-catalyzed biosynthesis of CWPS remains unclear, especially at the molecular level. This actually affects the investigation of the relationships between the structure and function. Clarifying the function of CWPS-related genes will enrich our knowledge on high-value enzymes for their artificial synthesis and optimization *in vitro*. In this review, we summarize the biosynthesis pathways of cell wall polysaccharides in seaweeds and provide a better understanding of their regulatory mechanism.

## Alginate

In brown seaweeds, cellulose only accounts for 1–8% of total dry weight of the brown seaweeds, whereas the anionic polysaccharides, namely alginate and fucoidan are the main cell wall components (Cronshaw et al., 1958; Kloareg and Quatrano, 1988). Alginate and fucoidan are predominantly extracted from the brown seaweeds (Moradali et al., 2018). Alginate is one linear polysaccharide composed of β-(1-4)-linked D-mannuronic acid (M) and α-L-guluronic acid (G). The M/G ratio and the block composition affect properties of alginate, thus providing either rigidity or flexibility to different tissues of the kelp. The initial investigation of enzymes involved in alginate biosynthesis was focused on mannuronan C-5 epimerase (MC5E), which is responsible for the conversion of M into G residues at the polymer level (Haug and Larsen, 1969). Rødde et al. (1993) measured MC5E activity in *Laminaria digitata*, and found that the synthesis of MC5E in the kelp protoplasts was essential for the new cell wall formation. Nyvall et al. (2003) summarized the pathway of alginate biosynthesis in brown algae, based on the biochemical analysis of the first five steps in *Fucus gardneri* and the cloning of six MC5E full-length coding sequences from *L. digitata*. Within expressed sequence tags (ESTs) dataset, 22 different MC5Es were identified from the cell wall biosynthesis genes (Roeder et al., 2005). The upregulation of MC5E transcripts during protoplast regeneration and sporophyte elicitation enabled *L. digitata* to rapidly modify its cell wall in response to marine environmental variations (Tonon et al., 2008).

So far, seven brown seaweed species had complete or draft genome sequences released, including *Ectocarpus siliculosus* (Cock et al., 2010; Cormier et al., 2017), *Saccharina japonica* (Ye et al., 2015), *Cladosiphon okamuranus* (Nishitsuji et al., 2016, 2020), *Nemacystus decipiens* (Nishitsuji et al., 2019), *Macrocystis pyrifera* (NCBI Bioproject: PRJNA605694), *Sargassum fusiforme* (Wang et al., 2020), and *Undaria pinnatifida* (Shan et al., 2020; Graf et al., 2021). The alginate- and fucoidan-relevant genes in these genomes are listed in Supplementary Table 1. The alginate-specific steps in *Ectocarpus* were proposed to be acquired by horizontal gene transfer (HGT) from an actinobacterium (Michel et al., 2010). Chi et al. (2018) found that the rise of the alginate pathway had complex endosymbiotic gene transfer (EGT) origins. Except for providing insights into the origin and evolution of alginate-related genes, these genomic datasets have also enabled the deeper understanding of the regulatory mechanism of alginate biosynthesis. Fischl et al. (2016) generated the first recombinant and active MC5E from brown algae. Subsequently, two soluble MC5Es from *S. japonica* have been proven active in the conversion of M into G (Inoue et al., 2016; Zhang et al., 2021). Tenhaken et al. (2011) isolated a candidate GDP-mannose dehydrogenase (GMD) in the *E. siliculosus* genome and found that Na_2_SO_4_, NaCl and KCl led to an increase in enzymatic activity. In *S. japonica*, Mg^2+^ could activate two SjGMDs potentially by improving the binding of substrate (Zhang et al., 2016). Subsequently, Zhang et al. (2018) found that the maximum activity of phosphomannomutase/phosphoglucomutase (PMM/PGM) occurred with the presence of Mg^2+^. Chi et al. (2018) reported that divalent ions of Mg^2+^, Mn^2+^, Ca^2+^, and Cu^2+^ promoted the activity of mannose-1-phosphate guanylyltransferase (MPG). Moreover, the transcriptomic and metabolic analysis revealed that higher expression of alginate biosynthetic genes in *Saccharina* sporophytes might be important for the increased thallus strength and toughness (Chi et al., 2018). Shao et al. (2019) identified candidate genes responsible for the high content of alginate in the distal blade of *S. japonica*.

## Fucoidan

Fucoidan is a sulfated polysaccharide containing α-(1→3) or α-(1→4)-linked L-fucose, which mainly exists in the cell wall matrix of brown algae and is not found in land plants. In Laminariales species, the concentration and structure of fucoidans vary with reproduction, tissue position, season and environmental factors (Bruhn et al., 2017). The maximum amount of fucoidan was accumulated during reproduction season in *S. japonica*, *Sargassum pallidum*, and *Stephanocystis crassipes*, but was not highly correlated with sea water temperature, salinity and biogenic elements (Skriptsova, 2016). The sulfation and molecular weight of fucoidan influenced its biological activity (Puri et al., 2022). Due to its anticoagulant, antimutagenic, immunostimulatory and antioxidant properties, the fucoidan is predominantly used in pharmaceuticals and therapeutics (Patel, 2012; Puri et al., 2022). Michel et al. (2010) first reported the fucoidan biosynthesis pathway in *E. siliculosus*, and proposed the *de novo* pathway catalyzed by GDP-mannose 4,6-dehydratase (GM46D) and GDP-fucose synthetase (GFS), and the salvage pathway with the involvement of fucokinase (FK) and GDP-fucose pyrophosphorylase (GFPP). Ye et al. (2015) compared the carbohydrate metabolism pathways based on *S. japonica* and 14 other algal genomes, and found that brown algae and diatoms harbor the complete fucoidan biosynthesis pathway. Unlike *E. siliculosus*, one fused FK-GFPP gene encoding a bifunctional enzyme possessing both L-fucokinase and GDP-fucose pyrophosphorylase activities was identified in the genomes of *C. okamuranus* and *N. decipiens* (Nishitsuji et al., 2016, 2019). Supplementary Table 1 lists the comparison of fucoidan pathway in distinct brown algal genomes. In addition, twenty-seven UDP-D-xylose: L-fucose-α-1,3-D-xylosyltransferases (FucXylTs) specifically catalyzed D-xylose to fucose were identified in brown algae (Han et al., 2019). Lu et al. (2020) screened 104 fucoidan-relevant genes from *S. japonica* and investigated the structure and transcriptional profiles in response to abiotic stress for sulfotransferase (ST) genes. However, the function of ST remains unclear ascribing to the absence of biochemical verification of this enzyme.

## Agar

**FIGURE 1:**
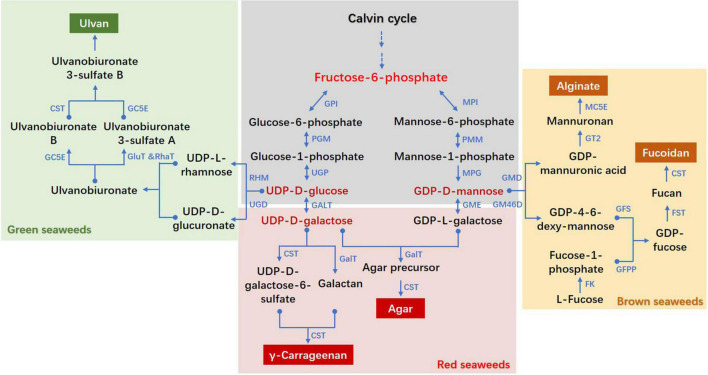
Proposed biosynthesis pathways of cell wall polysaccharides in seaweeds. CST, carbohydrate sulfotransferase; FK, fucokinase; FST, fucosyltransferase; GalT, galactosyltransferase; GALT, galactose-1-phosphate uridylyltransferase; GC5E, glucuronyl C5-epimerase; GFS, GDP-fucose synthetase; GFPP, GDP-fucose pyrophosphorylase; GluT, glucuronyltransferase; GM46D, GDP-mannose 4,6-dehydrogenase; GMD, GDP-mannose dehydrogenase; GME, GDP-mannose-3′,5′-epimerase; GPI, glucose-6-phosphate isomerase; GT, glycosyltransferase; MC5E, mannuronate C5-epimerase; MPI, mannose-6-phosphate isomerase; MPG, mannose-1-phosphate guanylyltransferase; PGM, phosphoglucomutase; PMM, phosphomannomutase; RhaT, α-1,4-rhamnosyltransferase; RHM, rhamnose synthase; UGD, UDP-D-glucose dehydrogenase; UGP, UTP-glucose-1-phosphate uridylyltransferase. The pathways referred to diverse literatures: (Lahaye and Robic, 2007; Collén et al., 2014; Ficko-Blean et al., 2015; Lee et al., 2017; Chi et al., 2018; Ho et al., 2018; Shao et al., 2019; Lipinska et al., 2020).

The hydrocolloids galactan agar is a water-soluble, gel-forming CWPS in red seaweeds and is widely used in food, pharmaceutical and biotechnology fields. The major agarophytes are *Gracilaria*, *Curdiea*, *Hydropuntia*, *Gelidium*, and *Pterocladia* (Sasuga et al., 2017). The agar biosynthesis was poorly understood and the hypothetical pathway was mainly deduced from chemical analysis in land plants and red seaweeds (Collén et al., 2004). Lee et al. (2017) proposed an agar biosynthetic pathway that starts from fructose-6-phosphate (F6P), which is then catalyzed to UDP-D-galactose (by galactose-1-phosphate uridylyltransferase, GALT) and GDP-L-galactose (by GDP-mannose-3,5-epimerase, GME) to form agar precursor chain. Figure 1 shows the schematic diagram of agar biosynthesis. Prior to this hypothetical pathway, GALT and GME were individually verified to have a regulatory role on the content of agar. The cloning and structure analysis of *GALT* has been reported in *Gracilaria gracilis* (Lluisma and Ragan, 1998) and *Gracilariopsis lemaneiformis* (Li et al., 2010). The *GALT* and *GME* transcripts and enzyme activities were found to be highest in *G. changii* and lowest in *G. salicornia*, corresponding to their respective agar yields (Siow et al., 2012, 2013). The relationship between agar content and expression levels of UDP-glucose pyrophosphorylase (UGP) gene in *G. lemaneiformis* indicated that UGP was a potential molecular marker to reflect the agar yields (Chang et al., 2014). Recently, Yu et al. (2021) identified one *GALT* gene from *Neoporphyra haitanensis*, and proposed that it might be derived from primary endosymbiotic eukaryotic hosts. Unlike GALT, GME and UGP, which are shared by many organisms, the galactosyl transferases were unique in agarophytes and were difficult to be identified. The *G. changii* genome annotated homologous genes for chondroitin sulfate synthases and chondroitin sulfate *N*-acetylgalactosaminyl transferase, which were regarded as potential galactosyl transferases for agar biosynthesis in *Gracilaria* (Ho et al., 2018). In addition, two carbohydrate sulfotransferase (CST) genes belonging to the sulfotransferase subfamily 2 were believed to be candidate genes for agar sulfotransferases (Ho et al., 2018). Nonetheless, the function of these presumed genes in *Gracilaria* awaits further investigation.

## Carrageenan

Carrageenans are commercially extracted from *Kappaphycus* and *Eucheuma*, and are dominantly produced in Indonesia, the Philippines and Malaysia (Porse and Rudolph, 2017). Usually, carrageenan can be used as additives in food, beverage, agriculture and animal feed and so forth. Molecular study on carrageenan biosynthesis remains very limited. To date, the only biochemically characterized enzymes in this pathway are galactose sulfurylases (Genicot-Joncour et al., 2009; Lipinska et al., 2020). Within the *Chondrus* genome dataset, genes encoding carrageenan-relevant enzymes, including CST, glycosyltransferase (GT), glycoside hydrolase (GH16), and galactose-6-sulfurylase were identified (Collén et al., 2013, 2014). CSTs and GTs in carrageenan biosynthesis in *C. crispus* were closely related to those involved in the synthesis of sulfated animal sugars, implying an ancient eukaryotic origin for these pathways (Ficko-Blean et al., 2015). The findings of conserved CSTs in brown seaweeds and red seaweeds through convergent evolution, which were not found in land plants and freshwater algae, inferred a critical role of sulfated polysaccharides in adapting to high-salinity environment (Brawley et al., 2017). The differential gene expression of carrageenan-related genes in carrageenanophytes were reported. Song et al. (2014) identified 8 differentially expressed KEGG orthologs for sulfur metabolism which might be related to the biosynthesis of three types of carrageenans in *Betaphycus*, *Kappaphycus*, and *Eucheuma*. Differential expression of multigenic genes of *CSTs*, *GTs*, *GH* and galactose-sulfurylases supported that carrageenan biosynthesis played a crucial role in the physiological differentiation between the isomorphic life cycle stages of *C. crispus* (Lipinska et al., 2020).

## Ulvan

The CWPS in Ulvaceae species account for 38–54% of cell dry weight with a majority of water-soluble ulvan (Lahaye and Kaeffer, 1997). In green seaweeds, the genera *Ulva*, *Monostroma*, and *Gayralia* synthesize the highly anionic ulvan polysaccharides (Domozych et al., 2012). As a gelling sulfated polysaccharide, ulvan attracts significant interest in the fields of agriculture, human health, and biomaterials. It is a complex polyanionic heteropolysaccharide with sugar compositions of rhamnose, glucuronate, iduronate and xylose (Kidgell et al., 2019). Lahaye and Robic (2007) proposed pathways for the biosynthesis of these four nucleotide sugar precursors of ulvan. On this basis, an enzyme-catalyzed ulvan biosynthesis pathway was proposed but none of the enzyme activities were experimentally verified (Ficko-Blean et al., 2015). Sea lettuce genome released potential genes encoding cell wall-related protein, but the polysaccharide biosynthesis was not discussed (De Clerck et al., 2018). Although no CST homologs were found in *Chara* genome, these genes conserved with those in animals, brown seaweeds and red seaweeds were retrieved from *Ulva* genome (Nishiyama et al., 2018; Kloareg et al., 2021). Phylogenetic analysis revealed that these CSTs were lost in freshwater and land plants, which solidly supported the hypothesis that cell wall sulfated polysaccharides were lost in the green lineage as an adaption to sulfate-scarce freshwater and terrestrial environments (Kloareg et al., 2021). Based on the above research, Figure 1 displayed the presumed ulvan biosynthesis pathway, with a disaccharide unit of rhamnose and iduronate.

## Conclusion and Perspectives

The activity and function of CWPS depend on their structure composition, and the latter is affected by catalytic enzymes at each step of the metabolic pathway. In this review, we have summarized the molecular evidence supporting the presence of genes encoding enzymes responsible for the biosynthesis of alginate, fucoidan, agar, carrageenan and ulvan. Together with the previously proposed pathways of CWPS metabolism in seaweeds, which were predominantly deduced from *in vivo* enzyme isolation and chemistry analysis, we constructed a schematic diagram of cell wall polysaccharide biosynthesis pathways with F6P as a common upstream metabolite (Figure 1). There are three important intermediate metabolites, GDP-D-mannose, UDP-D-glucose and UDP-D-galactose. GDP-D-mannose is the last shared metabolite for alginate, fucoidan and agar/carrageenan, whereas UDP-D-glucose is a common upstream metabolite for ulvan and agar/carrageenan. From UDP-D-galactose, agar or carrageenan is individually synthesized in different red algal species. The corresponding branching enzymes of GMD, GM46D, GME, UGD, and GALT therefore play important roles in the synthesis of specific CWPS in each seaweed. Fucoidan, agar, carrageenan and ulvan are sulfated polysaccharides, of which CSTs are key enzymes for their structure composition. In the last decade, the mining of CWPS-related genes has rapidly progressed ascribing to the completion of a series of multicellular algal genomes. Gene origin and evolution through phylogenetic analysis was the research focus, with a few genes’ heterologous expression and recombinant enzyme kinetic analysis. However, functional verification of corresponding genes was lagging due to the lack of reverse genetic manipulation and effective gene editing tools in macroalgae. Intensive study and functional verification of CWPS genes are highly needed to further clarify their biosynthesis pathways in seaweeds.

## Author Contributions

ZS and DD conceptualized the manuscript. ZS wrote the draft manuscript. DD reviewed and revised the manuscript. Both authors approved the final submitted version.

## Conflict of Interest

The authors declare that the research was conducted in the absence of any commercial or financial relationships that could be construed as a potential conflict of interest.

## Publisher’s Note

All claims expressed in this article are solely those of the authors and do not necessarily represent those of their affiliated organizations, or those of the publisher, the editors and the reviewers. Any product that may be evaluated in this article, or claim that may be made by its manufacturer, is not guaranteed or endorsed by the publisher.

## References

[B1] Baldauf S. L. (2008). An overview of the phylogeny and diversity of eukaryotes. *J. Syst. Evol.* 46 263–273. 10.3724/SP.J.1002.2008.08060

[B2] Brawley S. H. Blouin N. A. Ficko-Blean E. Wheeler G. L. Lohr M. Goodson H. V. (2017). Insights into the red algae and eukaryotic evolution from the genome of *Porphyra umbilicalis* (*Bangiophyceae*, *Rhodophyta*). *Proc. Natl. Acad. Sci. U.S.A.* 114 E6361–E6370. 10.1073/pnas.1703088114 28716924 PMC5547612

[B3] Bruhn A. Janicek T. Manns D. Nielsen M. M. Balsby T. J. S. Meyer A. S. (2017). Crude fucoidan content in two North Atlantic kelp species, *Saccharina latissimi* and *Laminaria digitata*-seasonal variation and impact of environmental factors. *J. Appl. Phycol.* 29 3121–3137. 10.1007/s10811-017-1204-5 29213185 PMC5705760

[B4] Cai J. Lovatelli A. Aguilar-Manjarrez J. Cornish L. Dabbadie L. Desrochers A. (2021). *Seaweeds and Microalgae: An Overview for Unlocking Their Potential in Global Aquaculture Development.* Rome: FAO Fisheries and Aquaculture Circula, 1229. 10.4060/cb5670en

[B5] Campbell I. Macleod A. Sahlmann C. Neves L. Funderud J. Øverland M. (2019). The environmental risks associated with the development of seaweed farming in Europe–prioritizing key knowledge gaps. *Front. Mar. Sci.* 6:107. 10.3389/fmars.2019.00107

[B6] Chang L. P. Sui Z. H. Fu F. Zhou W. Wang J. G. Kang K. H. (2014). Relationship between gene expression of UDP-glucose pyrophosphorylase and agar yield in *Gracilariopsis lemaneiformis* (*Rhodophyta*). *J. Appl. Phycol.* 26 2435–2441. 10.1007/s10811-014-0277-7

[B7] Chi S. Liu T. Wang X. M. Wang R. Wang S. S. Wang G. L. (2018). Functional genomics analysis reveals the biosynthesis pathways of important cellular components (alginate and fucoidan) of Saccharina. *Curr. Genet.* 64 259–273. 10.1007/s00294-017-0733-4 28825126 PMC5778160

[B8] Cock J. M. Sterck L. Rouzé P. Scornet D. Allen A. E. Amoutzias G. (2010). The Ectocarpus genome and the independent evolution of multicellularity in brown algae. *Nature* 465 617–621. 10.1038/nature09016 20520714

[B9] Coelho S. M. Cock J. M. (2020). Brown algal model organisms. *Annu. Rev. Genet.* 54 71–92. 10.1146/annurev-genet-030620 33228413

[B10] Collén J. Cornish M. L. Craigie J. Ficko-Blean E. Hervé C. Krueger-Hadfield S. A. (2014). “Chondrus crispus– A present and historical model organism for red seaweeds,” in *Advances in Botanical Research*, 71 ed. Bourgougnon N. (Amsterdam: Elsevier Ltd), 53–89. 10.1016/B978-0-12-408062-1.00003-2

[B11] Collén J. Porcel B. Carré W. Ball S. G. Chaparro C. Tonon T. (2013). Genome structure and metabolic features in the red seaweed *Chondrus crispus* shed light on evolution of the Archaeplastida. *Proc. Natl. Acad. Sci. U.S.A.* 110 5247–5252. 10.1073/pnas.1221259110 23503846 PMC3612618

[B12] Collén P. N. Camitz A. Hancock R. D. Viola R. Pedersen M. (2004). Effect of nutrient deprivation and resupply on metabolites and enzymes related to carbon allocation in *Gracilaria tenuistipitata* (*Rhodophyta*). *J. Phycol.* 40 305–314. 10.1111/j.1529-8817.2004.02174

[B13] Cormier A. Avia K. Sterck L. Derrien T. Wucher V. Andres G. (2017). Re-annotation, improved large-scale assembly and establishment of a catalogue of noncoding loci for the genome of the model brown alga *Ectocarpus*. *New Phytol.* 214 219–232. 10.1111/nph.14321 27870061

[B14] Cronshaw J. Myers A. Preston R. D. (1958). A chemical and physical investigation of the cell walls of some marine algae. *Biochim. Biophys. Acta* 27 89–103. 10.1016/0006-3002(58)90295-6 13510254

[B15] De Clerck O. Kao S. M. Bogaert K. A. Blomme J. Foflonker F. Kwantes M. (2018). Insights into the evolution of multicellularity from the sea lettuce genome. *Curr. Biol.* 28 2921–2933. 10.1016/j.cub.2018.08.015 30220504

[B16] Domozych D. S. (2016). “Biosynthesis of the cell walls of the algae,” in *The Physiology of Microalgae*, eds Borowitzka M. A. Beardall J. Raven J. A. (Switzerland: Springer International Publishing), 47–63. 10.1007/978-3-319-24945-2_2

[B17] Domozych D. S. Ciancia M. Fangel J. U. Mikkelsen M. D. Ulvskov P. Willats W. G. T. (2012). The cell walls of green algae: a journey through evolution and diversity. *Front. Plant Sci.* 3:82. 10.3389/fpls.2012.00082 22639667 PMC3355577

[B18] FAO (2021). *Global Seaweed and Microalgae Production, 1950-2019. World Aquaculture Performance Indicators (WAPI) Factsheet.* Rome: Food and Agriculture Organization.

[B19] Ficko-Blean E. Hervé C. Michel G. (2015). Sweet and sour sugars from the sea: the biosynthesis and remodeling of sulfated cell wall polysaccharides from marine macroalgae. *Persp. Phycol.* 2 51–64. 10.1127/pip/2015/0028 25280126

[B20] Fischl R. Bertelsen K. Gaillard F. Coelho S. Michel G. Klinger M. (2016). The cell-wall active mannuronan C5-epimerases in the model brown alga Ectocarpus: From gene context to recombinant protein. *Glycobiology* 26 973–983. 10.1093/glycob/cww040 27026155

[B21] Genicot-Joncour S. Poinas A. Richard O. Potin P. Rudolph B. Kloareg B. (2009). The cyclization of the 3,6-anhydrogalactose ring of iota-carrageenan is catalyzed by two D-galactose-2,6-sulfurylases in the red alga *Chondrus crispus*. *Plant. Physiol.* 151 1609–1616. 10.1104/pp.109.144329 19734263 PMC2773109

[B22] Graf L. Shin Y. Yang J. H. Choi J. W. Hwang I. K. Nelson W. (2021). A genome-wide investigation of the effect of farming and human-mediated introduction on the ubiquitous seaweed *Undaria pinnatifida*. *Nat. Ecol. Evol.* 5 360–368. 10.1038/s41559-020-01378-9 33495590 PMC7929912

[B23] Guiry M. (2012). How many species of algae are there? *J. Phycol.* 48 1057–1063. 10.1111/j.1529-8817.2012.01222.x 27011267

[B24] Guiry M. D. Guiry G. M. (2014). *AlgaeBase. World-Wide Electronic Publication.* Galway: National University of Ireland.

[B25] Han W. T. Fan X. Teng L. H. Kaczurowski M. J. S. Zhang X. W. Xu D. (2019). Identification, classification, and evolution of putative xylosyltransferases from algae. *Protoplasma* 256 1119–1132. 10.1007/s00709-019-01358-2 30941581

[B26] Haug A. Larsen B. (1969). Biosynthesis of alginate. Epimerisation of D-mannuronic to L-guluronic acid residues in the polymer chain. *Biochim. Biophy. Acta* 192 557–559. 10.1016/0304-4165(69)90414-0 5368261

[B27] Ho C.-L. Lee W. Lim E.-L. (2018). Unraveling the nuclear and chloroplast genomes of an agar producing red macroalga. *Genomics* 110 124–133. 10.1016/j.ygeno.2017.09.003 28890206

[B28] Inoue A. Satoh A. Morishita M. Tokunaga Y. Miyakawa T. Tanokura M. (2016). Functional heterologous expression and characterization of mannuronan C5-epimerase from the brown alga *Saccharina japonica*. *Algal. Res.* 16 282–291. 10.1016/j.algal.2016.03.030

[B29] Kidgell J. T. Magnusson M. M. de Nys R. Glasson C. R. K. (2019). Ulvan: A systematic review of extraction, composition and function. *Algal. Res.* 39:101422. 10.1016/j.algal.2019.101422

[B30] Kloareg B. Badis Y. Cock J. M. Michel G. (2021). Role and evolution of the extracellular matrix in the acquisition of complex multicellularity in Eukaryotes: A macroalgal perspective. *Genes* 12:1059. 10.3390/genes12071059 34356075 PMC8307928

[B31] Kloareg B. Quatrano R. S. (1988). Structure of the cell walls of marine algae and ecophysiological functions of the matrix polysaccharides. *Oceanogr. Mar. Biol.* 26 259–315.

[B32] Lahaye M. Kaeffer B. (1997). Seaweed dietary fibres: Structure, physico-chemical and biological properties relevant to intestinal physiology. *Sci. Aliment*. 17 563–584.

[B33] Lahaye M. Robic A. (2007). Structure and functional properties of ulvan, a polysaccharide from green seaweeds. *Biomacromolecules* 8 1765–1774. 10.1021/bm061185q 17458931

[B34] Leandro A. Pereira L. Gonçalves A. M. M. (2020). Diverse applications of marine macroalgae. *Mar. Drugs* 18:17. 10.3390/md18010017 31878264 PMC7024196

[B35] Lee W.-K. Lim Y.-Y. Leow A. T.-C. Namasivayam P. Abdullah J. O. Ho C.-L. (2017). Biosynthesis of agar in red seaweeds: A review. *Carbohyd. Polym.* 164 23–30. 10.1016/j.carbpol.2017.01.078 28325321

[B36] Li M. Sui Z. H. Kang K. H. Zhang X. C. Zhu M. Yan B. (2010). Cloning and analysis of the galactose-1-phosphate uridylyltransferase (galt) gene of Gracilariopsis lemaneiformis (*Rhodophyta*) and correlation between gene expression and agar synthesis. *J. Appl. Phycol.* 22 157–164. 10.1007/s10811-009-9435-8

[B37] Lipinska A. P. Collén J. Krueger-Hadfield S. A. Mora T. Ficko-Blean E. (2020). To gel or not to gel: differential expression of carrageenan-related genes between the gametophyte and tetasporophyte life cycle stages of the red alga *Chondrus crispus*. *Sci. Rep. UK.* 10:11498. 10.1038/s41598-020-67728-6 32661246 PMC7359372

[B38] Lluisma A. O. Ragan M. A. (1998). Characterization of a galactose-1-phosphate uridylyltransferase gene from the marine red alga *Gracilaria gracilis*. *Curr. Genet.* 34 112–119. 10.1007/s002940050374 9724413

[B39] Lu C. Shao Z. R. Zhang P. Y. Duan D. L. (2020). Genome-wide analysis of the *Saccharina japonica* sulfotransferase genes and their transcriptional profiles during whole developmental periods and under abiotic stresses. *BMC Plant. Biol.* 20:271. 10.1186/s12870-020-02422-3 32527219 PMC7291590

[B40] Michel G. Tonon T. Scornet D. Cock J. M. Kloareg B. (2010). The cell wall polysaccharide metabolism of the brown alga *Ectocarpus siliculosus*. Insights into the evolution of extracellular matrix polysaccharides in Eukaryotes. *New Phytol.* 188 82–97. 10.1111/j.1469-8137.2010.03374.x 20618907

[B41] Moradali M. F. Ghods S. Rehm B. H. A. (2018). “Alginate biosynthesis and biotechnological production,” in *Alginates and Their Biomedical Applications*, eds Rehm B. Moradali M. (Berlin: Springer Series in Biomaterials Science and Engineering), 10.1007/978-981-10-6910-9_1

[B42] Nishitsuji K. Arimoto A. Higa Y. Mekaru M. Kawamitsu M. Satoh N. (2019). Draft genome of the brown alga. *Sci. Rep.* 9 4607–4611. 10.1038/s41598-019-40955-2 30872679 PMC6418280

[B43] Nishitsuji K. Arimoto A. Iwai K. Sudo Y. Hisata K. Fujie M. (2016). A draft genome of the brown alga, *Cladosiphon okamuranus*, S-strain: a platform for future studies of “mozuku” biology. *DNA Res.* 23 561–570. 10.1093/dnares/dsw039 27501718 PMC5144679

[B44] Nishitsuji K. Arimoto A. Yonashiro Y. Hisata K. Fujie M. Kawamitsu M. (2020). Comparative genomics of four strains of the edible brown alga, *Cladosiphon okamuranus*. *BMC Genomics* 21:422. 10.1186/s12864-020-06792-8 32586267 PMC7318753

[B45] Nishiyama T. Sakayama H. de Vries J. Buschmann H. Saint-Marcoux D. Ullrich K. K. (2018). The Chara genome: secondary complexity and implications for plant terrestrialization. *Cell* 174 448–464. 10.1016/j.cell.2018.06.033 30007417

[B46] Nyvall P. Corre E. Boisset C. Barbeyron T. Rousvoal S. Scornet D. (2003). Characterization of mannuronan C-5-epimerase genes from the brown alga *Laminaria digitata*. *Plant Physiol.* 133 726–735. 10.1104/pp.103.025981 14526115 PMC219047

[B47] Ortega A. Geraldi N. R. Alam I. Kamau A. A. Acinas S. G. Logares R. (2019). Important contribution of macroalgae to oceanic carbon sequestration. *Nat. Geosci.* 12 748–754. 10.1038/s41561-019-0421-8

[B48] Patel S. (2012). Therapeutic importance of sulfated polysaccharides from seaweeds: updating the recent findings. *3Biotech* 2 171–185. 10.1007/s13205-012-0061-9

[B49] Porse H. Rudolph B. (2017). The seaweed hydrocolloid industry: 2016 updates, requirements, and outlook. *J. Appl. Phycol.* 29 2187–2200. 10.1007/s10811-017-1144-0

[B50] Puri M. Gupta A. McKinnon R. A. Abraham R. E. (2022). Marine bioactives: from energy to nutrition. *Trends Biotechnol.* 40 271–280. 10.1016/j.tibtech.2021.08.004 34507810

[B51] Rhein-Knudsen N. Meyer A. S. (2021). Chemistry, gelation, and enzymatic modification of seaweed food hydrocolloids. *Trends Food. Sci. Techn.* 109 608–621. 10.1016/j.tifs.2021.01.052

[B52] Rioux L.-E. Turgeon S. L. (2015). “Seaweed carbohydrates,” in *Seaweed Sustainability-Food and Non-Food Applications*, eds Tiwari B. K. Troy D. J. (Elsevier: Academic Press), 141–192. 10.1016/B978-0-12-418697-2.00007-6

[B53] Rødde R. S. H. Østgaard K. Larsen B. A. (1993). Mannuronan C-5 epimerase activity in protoplasts of *Laminaria digitata*. *Hydrobiologia* 260 577–581. 10.1007/BF00049073

[B54] Roeder V. Collén J. Rousvoal S. Corre E. Leblanc C. Boyen C. (2005). Identification of stress gene transcripts in *Laminaria digitata* (*Phaeophyceae*) protoplast cultures by expressed sequence tag analysis. *J. Phycol.* 41 1227–1235. 10.1111/j.1529-8817.2005.00150.x

[B55] Sasuga K. Yamanashi T. Nakayama S. Ono S. Mikami K. Tomoya Y. (2017). Optimization of yield and quality of agar polysaccharide isolated from the marine red macroalga Pyropia yezoensis. *Algal. Res.* 26 123–130. 10.1016/j.algal.2017.07.010

[B56] Shahbandeh M. (2021). *Value of the Seaweed Market Worldwide 2020-2028.* Hamburg: Statista.

[B57] Shan T. F. Yuan J. B. Su L. Li J. Leng X. F. Zhang Y. (2020). First genome of the brown alga *Undaria pinnatifida*: chromosome-level assembly using PacBio and Hi-C technologies. *Front. Genet.* 11:140. 10.3389/fgene.2020.00140 32184805 PMC7058681

[B58] Shao Z. R. Zhang P. Y. Lu C. Li S. Chen Z. H. Wang X. L. (2019). Transcriptome sequencing of *Saccharina japonica* sporophytes during whole developmental periods reveals regulatory networks underlying alginate and mannitol biosynthesis. *BMC Genomics* 20:975. 10.1186/s12864-019-6366-x 31830918 PMC6909449

[B59] Siow R. S. Teo S. S. Ho W. Y. Shukor M. Y. B. A. Phang S. M. Ho C. L. (2012). Molecular cloning and biochemical characterization of galactose-1-phosphate uridylyltransferase from *Gracilaria changii* (*Rhodophyta*). *J. Phycol.* 48 155–162. 10.1111/j.1529-8817.2011.01105.x 27009660

[B60] Siow R. S. Teoh S. Teo S. S. Shukor M. Y. B. A. Phang S. M. Ho C. L. (2013). Molecular cloning and characterization of GDP-mannose-3’, 5’-epimerase from *Gracilaria changii*. *J. Appl. Phycol.* 25 1309–1318. 10.1007/s10811-013-9987-5

[B61] Skriptsova A. V. (2016). Seasonal variations in the fucoidan content of brown algae from Peter the Great Bay. *J. Mar. Biol*. 42 351–356. 10.1134/S1063074016040106

[B62] Song L. P. Wu S. X. Sun J. Wang L. Liu T. Chi S. (2014). De novo sequencing and comparative analysis of three red algal species of family Solieriaceae to discover putative genes associated with carrageenan biosynthesis. *Acta Oceanol. Sin.* 33 45–53. 10.1007/s13131-014-0440-7

[B63] Tenhaken R. Voglas E. Cock J. M. Neu V. Huber C. G. (2011). Characterization of GDP-mannose dehydrogenase from the brown alga *Ectocarpus siliculosus* providing the precursor for the alginate polymer. *J. Biol. Chem.* 286 16707–16715. 10.1074/jbc.M111.230979 21454608 PMC3089512

[B64] Tonon T. Rousvoal S. Roeder V. Boyen C. (2008). Expression profiling of the mannuronan C5-epimerase multigenic family in the brown alga *Laminaria digitata* (*Phaeophyceae*) under biotic stress conditions. *J. Phycol.* 44 1250–1256. 10.1111/j.1529-8817.2008.00580.x 27041721

[B65] Vincent A. Stanley A. Ring J. (2020). *Hidden Champion of the Ocean: Seaweed as a Growth Engine for a Sustainable European.Future.* London, UK: Seaweed for Europe.

[B66] Wang S. Q. Lin L. D. Shi Y. J. Qian W. G. Li N. Yan X. F. (2020). First draft genome assembly of the seaweed *Sargassum fusiforme*. *Front. Genet*. 11:590065. 10.3389/fgene.2020.590065 33193728 PMC7644962

[B67] Ye N. H. Zhang X. W. Miao M. Fan X. Zheng Y. Xu D. (2015). Saccharina genomes provide novel insight into kelp biology. *Nat. Commun.* 6:6986. 10.1038/ncomms7986 25908475 PMC4421812

[B68] Yong W. T. L. Thien V. Y. Rupert R. Rodrigues K. F. (2022). Seaweed: A potential climate change solution. *Renew. Sust. Energ. Rev.* 159:112222. 10.1016/j.rser.2022.112222

[B69] Yu Y. H. Jia X. L. Wang W. L. Jin Y. M. Liu W. Z. Wang D. M. (2021). Floridean starch and floridoside metabolic pathways of Neoporphyra haitanensis and their regulatory mechanism under continuous darkness. *Mar. Drugs* 19 664. 10.3390/md19120664 34940663 PMC8703398

[B70] Zhang P. Y. Lu C. Shao Z. R. Liu F. L. Yao J. T. Duan D. L. (2021). Genome-wide transcriptome profiling and characterization of mannuronan C5-epimerases in *Saccharina japonica*. *Algal. Res.* 60:102491. 10.1016/j.algal.2021.102491

[B71] Zhang P. Y. Shao Z. R. Jin W. H. Duan D. L. (2016). Comparative characterization of two GDP mannose dehydrogenase genes from *Saccharina japonica* (*Laminariales*, *Phaeophyceae*). *BMC Plant Biol.* 16:62. 10.1186/s12870-016-0750-3 26956020 PMC4782291

[B72] Zhang P. Y. Shao Z. R. Li L. Liu S. Yao J. T. Duan D. L. (2018). Molecular characterisation and biochemical properties of phosphomannomutase/phosphoglucomutase (PMM/PGM) in the brown seaweed *Saccharina japonica*. *J. Appl. Phycol.* 30 2687–2696. 10.1007/s10811-018-1460-z

